# The victim-bully cycle of sexual minority school adolescents in China: prevalence and the association of mood problems and coping strategies

**DOI:** 10.1017/S2045796020000918

**Published:** 2020-11-06

**Authors:** Yuanyuan Wang, Hui Yu, Yong Yang, Ronghua Li, Amanda Wilson, Shuilan Wang, Jack Drescher, Runsen Chen

**Affiliations:** 1 National Clinical Research Center for Mental Disorders, Department of Psychiatry, and China National Technology Institute on Mental Disorders, The Second Xiangya Hospital of Central South University, Changsha, 410011, Hunan, China; 2 Division of Psychology, Faculty of Health and Life Sciences, De Montfort University, Leicester, UK; 3 Suzhou Guangji Hospital, Affiliated Guangji Hospital of Soochow University, Soochow University, Suzhou, China; 4 Postdoctoral Program in Psychotherapy and Psychoanalysis, New York University, New York, NY, USA; 5 Department of Psychiatry, Columbia University, New York, NY, USA; 6 Department of Psychiatry, The Affiliated Brain Hospital of Nanjing Medical University, Nanjing, China

**Keywords:** Adolescents, bullying, coping strategies, LGB, sexual minority, victim-bully cycle

## Abstract

**Aims:**

Compared to their heterosexual peers, youth who identify as lesbian, gay or bisexual (LGB) tend to suffer higher rates of peer victimisation from bullying. However, studies of LGB adolescents' participation as bullies are scarce. We aimed to examine the possible association of sexual minority identity and the heightened risk of not only being bullied but bullying others as well. We also explored the effect of one's sexual identity on their involvement in bullying through the mediation of coping strategies and mood states.

**Methods:**

A total of 12 218 students were recruited from 18 secondary schools in China. The demographic information, positive and negative coping strategies, mood state (anxiety, depression and hypomania) and information related to bullying and being bullied were collected. Multinomial regression was used to assess the heightened risk of sexual minority groups in comparison to their heterosexual adolescents' counterparts. A structural equation model (SEM) was used to test the mediating role of coping strategy and mood state between one's sex, sexual identity and bullying experience.

**Results:**

Two trends could be observed: (1) LGB groups reported heightened risks of being bullied and bullying others at school than heterosexual peers. However, being a sexual-undeveloped girl seemed to have a protective effect on bullying-related problems. (2) Birth-assigned males were more likely to be bullied as well as bullying others at school when compared to birth-assigned females. SEM analysis revealed that being a sexual minority was directly associated with a higher frequency of being bullied (*B* = 0.16, 95% CI [0.10, 0.22], *p* < 0.001) but not bullying others (*B* = 0.02, 95% CI [−0.02, 0.06], *p* = 0.398) when compared to the heterosexual group. Negative coping, hypomania, anxiety and depression were associated with a higher frequency of being bullied, while positive coping was associated with a lower frequency of being bullied. Moreover, negative coping, hypomania and depression were associated with a higher frequency of bullying others, while positive coping was associated with a reduced likelihood of bullying others. In addition, being bullied and bullying others were significantly correlated in the SEM model.

**Conclusions:**

This novel research investigated the dynamic nature of the interaction between victim and bullying of LGB school adolescents in China, with a specific exploration of the psychological mechanism behind the pattern of being bullied and bullying others. School-level interventions aimed at teaching positive coping strategies to lower psychological distress are recommended to support sexual minority students.

## Introduction

Bullying has been defined as intentional and repeated aggressive behaviours that occur in power and imbalanced interpersonal relationships (Whitney and Smith, 1993; Olweus, 1994). Bullying is a highly stressful experience and victims can have numerous negative outcomes (Duong and Bradshaw, 2014). Peer bullying experienced by adolescents is correlated with severe mental health problems – including suicidal ideation, suicide attempts, non-suicidal self-injury, depression and anxiety – and can have long-term detrimental life consequence (Klomek *et al*., 2007; Drydakis, 2019; Li *et al*., 2019; Chen *et al*., 2020). Due to the marginalised sexual orientation, lesbian, gay and bisexual (LGB) adolescents are more likely to face noticeable challenges and adverse experiences, including the victim-bully cycle (Button *et al*., 2012).

### Bullying in sexual minority individuals

Studies across different countries have demonstrated that sexual minorities tend to be victims of verbal, physical and social bullying (Birkett *et al*., 2009; Robinson and Espelage, 2012; Collier *et al*., 2013; Robinson *et al*., 2013; Duong and Bradshaw, 2014). A previous meta-analysis showed that identifying as a sexual minority is a risk factor for suffering from bullying at school (Moyano and del Mar Sánchez-Fuentes, 2020). Researchers have consistently found that homophobic bullying is linked to negative mental health outcomes (Espelage *et al*., 2019; Peng *et al*., 2019). Much of the literature has focused on the experiences of sexual minority adolescents being bullied, while studies on sexual minority adolescents' participation in bullying are scarce. Previous research on the victim-bully cycle in middle school indicates that the relationship from bully to victim was reciprocal (Ma, 2001). Bully-victims can be defined as an individual who was a victim of bulling who then in turn reciprocates and bullies others (Swearer *et al*., 2001). Research on the victim and bully roles at school has found that the victim role was unstable and that the bully role was only moderately stable (Schäfer *et al*., 2005). Many studies have suggested the roles oscillate between bully and victim (Olweus, 1994; Slee, 1995; Swearer *et al*., 2001). The victim-bully cycle can be explained by social learning theory; the victims' bullying behaviours could be a socially learned behaviour as a response to coping with the situation (Ma, 2001). Bullying research has also found that some of the most extreme victims are also among the most aggressive of bullies (Perry *et al*., 1988). In addition, sexual minority youth who experience school bullying are more likely to engage in aggressive behaviours such as physical fights (Duong and Bradshaw, 2014). It is important to note that those who take on the role of both the victim and bully may be at the highest risk for depression and anxiety (Swearer *et al*., 2001).

### Bullying and mood problems

Research has shown that victims of bullying are at risk of mood disorders (Kochenderfer-Ladd and Skinner, 2002; Tenenbaum *et al*., 2011). There is ample documentation of the sexual minority population being at risk for depression and anxiety (D'augelli and Grossman, 2001; Meyer, 2003; Cochran and Mays, 2009; Bostwick *et al*., 2010; Chakraborty *et al*., 2011; Marshal *et al*., 2011; Fredriksen-Goldsen *et al*., 2013). Considering that sexual minority individuals have a higher likelihood of being bullied and a higher risk of having mood problems, bullying and mood disorders are likely to be prevalent in sexual minority adolescent at schools. Although the role of bullying within hypomania is not clear, a previous study suggests that bullying victimisation may increase the vulnerability of developing psychotic symptoms which could result in further hypomanic symptoms (Trotta *et al*., 2013). Sexual minority identity has been associated with psychosis (Pakula and Shoveller, 2013; Jacob *et al*., 2019), and researchers propose that stressful life events, such as bullying victimisation, may function as part of the underlying mechanism that results in hypomania (Jacob *et al*., 2019).

### Coping strategies and bullying

Many studies have investigated the association between coping strategies and bullying (Varjas *et al*., 2009; Tenenbaum *et al*., 2011). A previous longitudinal study in the USA found that bullying was more persistent for victims who applied negative coping strategies (i.e. fought back) than those who applied positive coping strategies (i.e. having a friend help) (Kochenderfer and Ladd, 1997). Researchers have indicated that victims of bullying generally found that the implementation of coping strategies was not effective in solving their problems (Tenenbaum *et al*., 2011). The application of adaptive coping strategies may help to prevent future bullying and victimisation (Varjas *et al*., 2009). Sexual minority adolescents with effective coping strategies are also less likely to experience adverse events such as being marginalised and being bullied (Kiperman *et al*., 2014).

### Hypothesis

To the best of our knowledge, no existing published research has investigated the associative variables on the interaction between bullying others and being bullied among sexual minority adolescents. Therefore, this study aimed to examine the influence of the sex orientation on bullying and being bullied through the mediation of coping strategies and mood states. It is hypothesised that engaging in positive coping strategies could reduce the risk of being bullied. Moreover, previous studies have noted that sex difference also leads to adolescents applying different coping strategies in response to bullying (Naylor *et al*., 2001). Thus, the current study will assess the influence of sex together with the influence of the sexual orientation as part of the bullying mechanism within the victim-bully cycle. This study also assessed the different odds ratio of sexual minority groups in comparison to heterosexual adolescents for a variety of bullying-related behaviours, in order to examine the disparities among the sexual minority groups.

## Methods

### Participants

Data were collected from 18 public secondary schools in Suzhou, a medium-size metropolitan city in China. Schoolteachers aided in the recruitment of subjects and it was made clear to potential subjects that participation was voluntary, and there were no adverse consequences for refusing to participate or for later if they chose to withdraw. A total of 12 354 questionnaires were completed and returned and the response rate was 83.2%. The study design and study participants have been described in a previous publication (Duan *et al*., 2020). All students (6688 boys and 5666 girls) answered the question about their birth-assigned sex; however, there were 43 boys and 73 girls who did not specify to which sex they were attracted to and were therefore excluded from further analysis. As such, the sample in the analysis consisted of 6625 birth-assigned boys (*M*_age_ = 14.94, s.d. = 1.48) and 5593 birth-assigned girls (*M*_age_ = 14.95, s.d. = 1.48).

The student's sexual orientation was measured by two questions (Denny *et al*., 2016): ‘What was your biological sex assigned at birth? (choose from male or female)’ and ‘Which sex are you attracted to? (choose from male, female, both, and none)’. Students were categorised into six groups based on their birth-assigned sex and sexual: Those boys and girls who were attracted to the opposite sex were classified as opposite-sex-attraction-boys and opposite-sex-attraction-girls, respectively. Birth-assigned males attracted to other males were categorised as same-sex-attraction-boys, while birth-assigned females attracted to females were categorised as same-sex-attraction-girls. Birth-assigned boys who reported being attracted to both sexes were designated as both-sex-attraction-boys and birth-assigned girls attracted to both sexes as both-sex-attraction-girls (Denny *et al*., 2016). As Fenaughty *et al*. (2019) point out, the choice of ‘to neither males nor females’ was not a particular measure of asexuality. Instead, we categorised the 2625 boys and 2395 girls who reported attracted to neither male nor female as sexual-undeveloped-boys and sexual-undeveloped-girls, because they are more likely to not yet experience sexual due to their young age rather than identifying as asexual. We believe these groups have meaningful differences, and a similar method of grouping among Chinese adolescents has been used to in a previous study (Huang *et al*., 2018).

### Measures

#### The Chinese Trait Coping Style Questionnaire

The Trait Coping Style Questionnaire (TCSQ) was used to measure positive and negative coping styles among the adolescents. There were 20 items, with ten items assessing each sub-scale. One example for positive coping was ‘I focus on the positive side and reappraise the situation’; and one example for negative coping was ‘If I had a confrontation with someone, I would cut the communication with the person for a long time’. Responses were rated on a 1–5 Likert scale from 1 (not me at all) to 5 (I do things completely this way). A higher composite score indicates a higher inclination to adopt the positive or negative coping style, respectively. The Cronbach's *α* was 0.85 for the positive coping style sub-scale, and 0.88 for the negative coping style in this sample.

#### The Patient Health Questionnaire

The Patient Health Questionnaire (PHQ-9) was used to measure the severity of depressive symptoms. There were nine items (e.g. ‘Little interest or pleasure in doing things’). Participants were asked to rate how often they had been bothered by any of the problems over the previous 2 weeks on a 0–3 point scale, where 0 = Not at all to 3 = Nearly every day. The Cronbach's *α* was 0.93 in this sample.

#### The 32-item Hypomania Checklist Chinese Version

The Chinese version of the Hypomania Checklist (HCL-32) was used to measure the level of hypomania in the adolescents. Participants were asked to choose yes or no on 32 statements about when they were in a state of ‘high spirit’. One example was ‘you are more talkative’. The Cronbach's *α* was 0.87 in this sample.

#### The Generalised Anxiety Disorder Screener

The Generalised Anxiety Disorder Screener (GAD-7) , which was previously validated in the general population, was used to measure anxiety symptoms; there are seven items (e.g. ‘worrying too much about different things’). Similar to the PHQ-9, participants were asked to report based on the previous 2 weeks' experiences, rated on a 0–3 point scale, where 0 = Not at all to 3 = Nearly every day. The Cronbach's *α* was 0.94 in this sample.

#### Frequency of being bullied at school

The frequency of being bullied at school was measured by five items. Participants were asked to rate their experience in the past academic year on a 1–4 point scale. Response options for this question were 0 = never, 1 = sometimes (1–2 per month), 2 = often (1–2 per week) and 3 = every day. One example was ‘Were you afraid of being kicked, pushed, or beaten at school?’ The total score of being bullied was calculated and the range of the composite score was between 0 and 15, with a higher score indicating a higher frequency of being bullied at school in the past academic year. The Cronbach's *α* was 0.68 in this sample. In the multinomial analyses, ratings of 1, 2 and 3 for each item were further grouped as a ‘yes’ response to the experience, in contrast to those who had not experienced bullying at school.

#### Frequency of bullying others at school

The frequency of bullying others at school was measured by five items. Participants were asked to rate their behaviours in the past academic year on a 1–4 point scale. Response options for this question were 0 = never, 1 = sometimes (1–2 per month), 2 = often (1–2 per week) and 3 = every day. One example was ‘Have you kicked, pushed, or beaten others at school?’ The total score of being bullied was calculated and the range of the composite score was between 0 and 15, with a higher score indicating a higher frequency of bullying others at school in the past academic year. The Cronbach's *α* was 0.66 in this sample. In the multinomial analyses, ratings of 1, 2 and 3 for each item were further grouped as a ‘yes’ response to the experience, in contrast to those who had experienced bullying at school.

### Analysis

All analyses were carried out using R software Mac version 3.6.1. Total numbers and valid percentages were calculated for each sexual orientation group. Given the large sample size of the survey, *p* < 0.01 was taken to indicate statistical significance in all analyses. To assess the difference in measured variables among the sex and sexual attraction groups, a MANOVA was conducted. To assess the increased risk of being bullied and bullying others, in association with different groups, a series of multinomial regression were conducted. To assess the psychological mechanism behind the association between sexual orientation and the frequency of being bullied as well as bullying others, a structural equation model (SEM) was constructed using R Lavaan package using negative and positive coping, hypomanic behavioural pattern, anxiety and depression as the mediators. The analysis incorporated several regression analyses simultaneously, also allowing correlations between theoretically related variables; in particular: negative and positive coping, anxiety and depression, as well as the frequency of being bullied and bullying others.

## Results

**Fig. 1.**
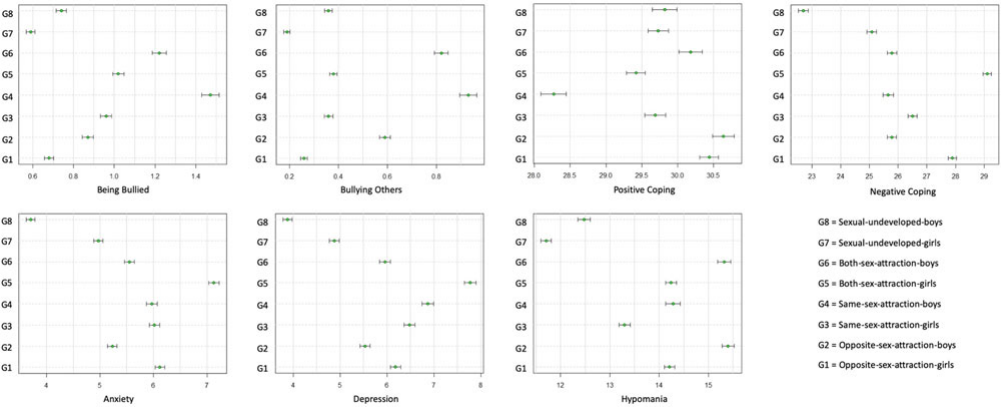
Centipede plot of group difference: means and 95% confidence interval of each sexual attraction group on study variables. *Note.* The mid-point indicates the mean and the bars indicate the 95% confidence interval. In each panel, any two groups with non-overlapping bars were significantly different on the measured variable.

The means and s.d.s of the variables used in the study by sexual attraction groups are shown in Table 1. A 2 × 4 MANOVA was conducted to test the potential effect of sex and sexual on all measured variables, including being bullied, bullying others, positive and negative coping, hypomania, anxiety and depression. There was a significant main effect of sex, *F*_(7, 10799)_ = 44.48, *p* < 0.001, Wilks' Λ = 0.97, partial *η*^2^ = 0.03, a significant main effect of sexual, *F*_(21, 31009)_ = 27.86, *p* < 0.001, Wilks' Λ = 0.95, partial *η*^2^ = 0.02, and a significant main interaction of sex and sexual, *F*_(21, 31009)_ = 6.04, *p* < 0.001, Wilks' Λ = 0.98, partial *η*^2^ = 0.01. As the interaction term was significant, follow-up *post hoc* comparisons were done by comparing the eight sexual groups. The results are summarised in Fig. 1, which indicates the estimated mean (the mid-point) and 95% confidence interval (the bars extended from the mid-point) for each group on all the measured variables. Any two groups with non-overlapping bars in each panel of the graph showed significant group difference.

**Table 1.** Means and s.d.s of study variables by sexual attraction

	*N*	%	Age	Positive Coping	Negative Coping	Hypomania	Anxiety	Depression	Being Bullied	Bullying Others
Opposite-sex-at-girl	1998	16.35%	15.42 (1.53)	30.44 (7.31)	27.89 (7.80)	14.21 (5.87)	6.12 (5.07)	6.18 (6.22)	0.68 (1.27)	0.26 (0.76)
Opposite-sex-at-boy	3136	25.67%	15.48 (1.56)	30.64 (8.45)	25.78 (8.71)	15.40 (6.94)	5.23 (5.02)	5.53 (6.02)	0.87 (1.50)	0.59 (1.23)
Same-sex-at-girl	274	2.24%	14.47 (1.27)	29.69 (8.17)	26.50 (9.25)	13.30 (6.29)	6.02 (5.42)	6.48 (6.66)	0.96 (1.53)	0.36 (1.00)
Same-sex-at-boy	212	1.74%	14.65 (1.62)	28.27 (9.97)	25.65 (10.20)	14.28 (8.26)	5.97 (5.66)	6.87 (7.16)	1.47 (2.43)	0.93 (1.94)
Both-sex-at-girl	926	7.58%	14.90 (1.49)	29.42 (7.39)	29.10 (8.26)	14.24 (6.19)	7.13 (5.49)	7.77 (7.03)	1.02 (1.52)	0.38 (0.86)
Both-sex-at-boy	652	5.34%	14.77 (1.40)	30.18 (9.20)	25.78 (9.34)	15.32 (7.65)	5.55 (5.39)	5.96 (6.50)	1.22 (1.96)	0.82 (1.58)
Sexual-undevelop-girl	2395	19.60%	14.64 (1.34)	29.73 (7.97)	25.08 (9.34)	11.71 (5.99)	4.97 (4.90)	4.88 (5.86)	0.59 (1.20)	0.19 (0.71)
Sexual-undevelop-boy	2635	21.57%	14.38 (1.10)	29.82 (9.62)	22.70 (9.48)	12.48 (6.98)	3.70 (4.48)	3.88 (5.37)	0.74 (1.39)	0.36 (0.88)
Total	12 218	100%	14.95 (1.48)	30.08 (8.45)	25.59 (8.89)	13.68 (6.75)	5.18 (5.07)	5.39 (6.13)	0.80 (1.45)	0.41 (1.03)

*Note.* at, attraction; undevelop, underdeveloped.

A series of multinomial logistic regressions were conducted to test whether being a sexual minority was associated with a greater risk of being bullied and a higher or lower likelihood of bullying others. The birth-assigned sex and sexual attraction category was used as the predictor, and the YES/NO answers to the being bullied or to bullying others were used as dependent variables (the opposite-sex-attraction-girls were treated as the reference group). Details of the ORs and effect sizes are summarised in Table 2; but two trends could be observed: (1) sexual minority groups reported heightened risks of being bullied and bullying others at school than heterosexual girls. However, being a sexual-undeveloped girl seemed to have a protective effect on bullying-related problems; and (2) birth-assigned males were more likely to be involved in being bullied as well as bullying others at school than birth-assigned females.

**Table 2.** Multinomial regression results indicating associations between sexual attraction and being bullied or bullying others

	*n* (No)	*n* (Yes)	Yes%	Odds ratio	95% CI of odds ratio	*Z*	*p*
Have you ever been bullied at school in this academic year?							
Opposite-sex-attraction-girl	1882	113	5.66%	1.00			
Opposite-sex-attraction-boy	2836	294	9.39%	1.73	[1.39, 2.16]	4.77	**<0.001**
Same-sex-attraction-girl	254	19	6.96%	1.24	[0.75, 2.08]	0.86	0.392
Same-sex-attraction-boy	167	41	19.71%	4.09	[2.77, 6.05]	7.06	**<0.001**
Both-sex-attraction-girl	863	63	6.80%	1.22	[0.90, 1.66]	1.20	0.229
Both-sex-attraction-boy	556	92	14.20%	2.76	[2.05, 3.67]	6.83	**<0.001**
Sexual-undeveloped-girl	2270	122	5.10%	0.90	[0.70, 1.15]	−0.83	0.409
Sexual-undeveloped-boy	2399	221	8.44%	1.53	[1.21, 1.93]	3.58	**<0.001**
Have you ever missed school because you were concerned about safety?							
Opposite-sex-attraction-girl	1822	171	8.58%	1.00			
Opposite-sex-attraction-boy	2918	205	6.56%	0.75	[0.61, 0.93]	−2.68	**0.007**
Same-sex-attraction-girl	242	29	10.70%	1.28	[0.84, 1,92]	1.15	0.249
Same-sex-attraction-boy	180	29	13.88%	1.72	[1.12, 2.64]	2.50	0.012
Both-sex-attraction-girl	809	114	12.35%	1.50	[1.17, 1.93]	3.17	**0.001**
Both-sex-attraction-boy	588	61	9.40%	1.10	[0.81, 1.51]	0.64	0.522
Sexual-undeveloped-girl	2239	150	6.28%	0.71	[0.57, 0.89]	−2.90	**0.004**
Sexual-undeveloped-boy	2473	136	5.21%	0.59	[0.47, 0.75]	−4.49	**<0.001**
Were you afraid of being kicked, pushed or beaten at school?							
Opposite-sex-attraction-girl	1768	213	10.75%	1.00			
Opposite-sex-attraction-boy	2775	333	10.71%	1.00	[0.84, 1.20]	−0.04	0.966
Same-sex-attraction-girl	226	45	16.61%	1.65	[1.16, 2.34]	2.81	**0.005**
Same-sex-attraction-boy	170	39	18.66%	1.90	[1.31, 2.74]	3.36	**<0.001**
Both-sex-attraction-girl	770	150	16.30%	1.62	[1.27, 2.05]	4.18	**<0.001**
Both-sex-attraction-boy	544	100	15.53%	1.53	[1.17, 1.95]	3.23	**0.001**
Sexual-undeveloped-girl	2160	218	9.17%	0.84	[0.68, 1.02]	−1.74	0.081
Sexual-undeveloped-boy	2350	249	9.58%	0.88	[0.72, 1.07]	−1.30	0.192
Have your belongings being stolen or damaged (e.g. books or bike)?							
Opposite-sex-attraction-girl	1668	325	16.31%	1.00			
Opposite-sex-attraction-boy	2342	786	25.13%	1.72	[1.49, 1.97]	7.42	**<0.001**
Same-sex-attraction-girl	208	65	23.81%	1.60	[1.20, 2.14]	3.05	**0.002**
Same-sex-attraction-boy	154	56	26.67%	1.86	[1.34, 2.58]	3.72	**<0.001**
Both-sex-attraction-girl	688	236	25.54%	1.76	[1.43, 2.14]	5.84	**<0.001**
Both-sex-attraction-boy	445	204	31.43%	2.35	[1.93, 2.89]	8.22	**<0.001**
Sexual-undeveloped-girl	2013	380	15.88%	0.97	[0.83, 1.14]	−0.38	0.701
Sexual-undeveloped-boy	2060	557	21.28%	1.39	[1.18, 1.63]	4.24	**<0.001**
Were you afraid of your belongings being stolen or damaged at school?							
Opposite-sex-attraction-girl	1619	376	18.85%	1.00			
Opposite-sex-attraction-boy	2458	670	21.42%	1.17	[1.02, 1.35]	2.22	0.026
Same-sex-attraction-girl	217	55	20.22%	1.09	[0.80, 1.49]	0.54	0.588
Same-sex-attraction-boy	160	50	23.81%	1.34	[0.97. 1.88]	1.73	0.084
Both-sex-attraction-girl	679	245	26.52%	1.55	[1.30, 1.86]	4.69	**<0.001**
Both-sex-attraction-boy	492	158	24.31%	1.38	[1.10, 1.72]	3.00	**0.003**
Sexual-undeveloped-girl	2006	386	16.14%	0.83	[0.70, 0.97]	−2.36	0.018
Sexual-undeveloped-boy	2157	461	17.61%	0.92	[0.79, 1.08]	−1.08	0.280
Have you ever bullied or laughed at others?							
Opposite-sex-attraction-girl	1754	241	12.08%	1.00			
Opposite-sex-attraction-boy	2366	755	24.19%	2.32	[1.97, 2.72]	10.48	**<0.001**
Same-sex-attraction-girl	229	43	15.81%	1.37	[0.96, 1.93]	1.74	0.082
Same-sex-attraction-boy	156	54	25.71%	2.52	[1.80, 3.49]	5.36	**<0.001**
Both-sex-attraction-girl	767	157	16.99%	1.49	[1.20, 1.86]	3.58	**<0.001**
Both-sex-attraction-boy	460	185	28.68%	2.93	[2.34, 3.60]	9.68	**<0.001**
Sexual-undeveloped-girl	2202	184	7.71%	0.61	[0.50, 0.75]	−4.83	**<0.001**
Sexual-undeveloped-boy	2217	392	15.02%	1.29	[1.07, 1.54]	2.87	**0.004**
Have you kicked, pushed, or beaten others at school?							
Opposite-sex-attraction-girl	1895	93	4.68%	1.00			
Opposite-sex-attraction-boy	2760	357	11.45%	2.64	[2.08, 3.32]	8.06	**<0.001**
Same-sex-attraction-girl	259	13	4.78%	1.02	[0.56, 1.84]	0.07	0.943
Same-sex-attraction-boy	177	33	15.71%	3.80	[2.46, 5.81]	6.14	**<0.001**
Both-sex-attraction-girl	868	57	6.16%	1.34	[0.96, 1.86]	1.68	0.093
Both-sex-attraction-boy	548	100	15.43%	3.72	[2.77, 4.95]	8.64	**<0.001**
Sexual-undeveloped-girl	2316	72	3.02%	0.63	[0.46, 0.86]	−2.85	**0.004**
Sexual-undeveloped-boy	2428	180	6.90%	1.51	[1.17, 1.93]	3.14	**0.002**
Have you stolen or damage others' belongings such as books or bikes?							
Opposite-sex-attraction-girl	1969	25	1.25%	1.00			
Opposite-sex-attraction-boy	3038	89	2.85%	2.31	[1.48, 3.63]	3.66	**<0.001**
Same-sex-attraction-girl	268	4	1.47%	1.18	[0.41, 3.39]	0.30	0.765
Same-sex-attraction-boy	196	15	7.11%	6.02	[3.13, 11.82]	5.36	**<0.001**
Both-sex-attraction-girl	907	15	1.63%	1.30	[0.68, 2.48]	0.80	0.421
Both-sex-attraction-boy	616	32	4.94%	4.09	[2.41, 6.96]	5.20	**<0.001**
Sexual-undeveloped-girl	2360	33	1.38%	1.10	[0.65, 1.88]	0.36	0.718
Sexual-undeveloped-boy	2561	53	2.03%	1.63	[1.02, 2.61]	2.00	0.046
Have you brought weapons (‘aggressive tools’ in Chinese) to school?							
Opposite-sex-attraction-girl	1920	68	3.42%	1.00			
Opposite-sex-attraction-boy	2927	204	6.52%	1.97	[1.49, 2.58]	4.73	**<0.001**
Same-sex-attraction-girl	259	14	5.13%	1.52	[0.84, 2.72]	1.40	0.160
Same-sex-attraction-boy	191	18	8.61%	2.66	[1.54, 4.62]	3.55	**<0.001**
Both-sex-attraction-girl	872	54	5.83%	1.75	[1.21, 2.53]	2.99	**0.003**
Both-sex-attraction-boy	595	53	8.18%	2.51	[1.73, 3.63]	4.88	**<0.001**
Sexual-undeveloped-girl	2319	70	2.93%	0.85	[0.61, 1.18]	−0.92	0.355
Sexual-undeveloped-boy	2506	107	4.09%	1.20	[0.90, 1.62]	1.18	0.237
Did you often get into fight at school in the past academic year?							
Opposite-sex-attraction-girl	1972	20	1.00%	1.00			
Opposite-sex-attraction-boy	2997	127	4.07%	4.18	[2.61, 6.68]	5.90	**<0.001**
Same-sex-attraction-girl	266	6	2.21%	2.22	[0.89, 5.58]	1.70	0.089
Same-sex-attraction-boy	196	15	7.11%	7.55	[3.78, 15.03]	5.78	**<0.001**
Both-sex-attraction-girl	915	9	0.97%	0.97	[0.44, 2.12]	−0.08	0.939
Both-sex-attraction-boy	607	41	6.33%	6.66	[3.86, 11.59]	6.85	**<0.001**
Sexual-undeveloped-girl	2365	23	0.96%	0.96	[0.55, 1.66]	−0.14	0.892
Sexual-undeveloped-boy	2544	69	2.64%	2.67	[1.56, 4.44]	3.85	**<0.001**

Bolded value <0.05, significant level was set at <0.05.

**Fig. 2.**
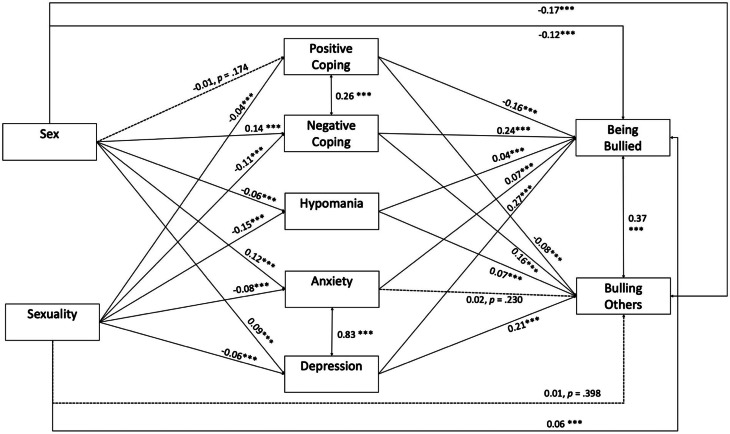
Final SEM model with the standardized coefficients labelled for each path. *Note.* ***p < 0.001. Sex: 0 = boys; 1 = girls. Sexuality: 0 = opposite-sex attraction; 1 = all other sexual preference.

An SEM (Fig. 2) was constructed to examine the mechanism behind the association between sex and sexual attraction and the frequency of being bullied and bullying behaviours, with positive and negative coping, hypomania, anxiety as well as depression as mediators. The results are summarised in Table 3. SEM analysis revealed that being a heterosexual girl was associated with a reduced risk of being bullied and bullying others in comparison to being a heterosexual boy. On the other hand, being a sexual minority was associated with a heightened risk of being bullied. Negative coping, hypomania, anxiety and depression were associated with a higher frequency of being bullied; while positive coping had a significant protective effect on the frequency of being bullied. Negative coping, hypomania and depression were associated with a higher frequency of bullying others, whereas positive coping was associated with a lower frequency of bullying others. Moreover, positive coping and negative coping were positively correlated, and so were anxiety and depression, as well as bullying others and being bullied.

**Table 3.** Summary of the SEM model

Model description	Path	Unstandardised coefficient	95% CI	*p*
Direct effect	Sex --> Being bullied	−0.33	[−0.39, −0.27]	**<0.001**
	Sex --> Bullying others	−0.34	[−0.38, −0.30]	**<0.001**
	Sexuality --> Being bullied	0.16	[0.10, 0.22]	**<0.001**
	Sexuality --> Bullying others	0.02	[−0.02, 0.06]	0.398
Mediators --> DV	Positive coping --> Being bullied	−0.03	[−0.034, −0.026]	**<0.001**
	Negative coping --> Being bullied	0.04	[0.036, 0.044]	**<0.001**
	Hypomania --> Being bullied	0.01	[0.006, 0.014]	**<0.001**
	Anxiety --> Being bullied	0.02	[0.01, 0.03]	**<0.001**
	Depression --> Being bullied	0.06	[0.05, 0.07]	**<0.001**
	Positive coping --> Bullying others	−0.01	[−0.012, −0.008]	**<0.001**
	Negative coping --> Bullying others	0.02	[0.018, 0.022]	**<0.001**
	Hypomania --> Bullying others	0.01	[0.006, 0.014]	**<0.001**
	Anxiety --> Bullying others	0.004	[−0.002, 0.010]	0.230
	Depression --> Bullying others	0.03	[0.024, 0.036]	**<0.001**
IV --> Mediators	Sex --> Positive coping	−0.22	[−0.53, 0.09]	0.174
	Sex --> Negative coping	2.51	[2.18, 2.84]	**<0.001**
	Sex --> Hypomania	−0.87	[−1.12, −0.62]	**<0.001**
	Sex --> Anxiety	1.26	[1.06, 1.46]	**<0.001**
	Sex --> Depression	1.12	[0.88, 1.36]	**<0.001**
	Sexuality --> Positive coping	−0.71	[−1.02, −0.40]	**<0.001**
	Sexuality --> Negative coping	−2.00	[−2.33, −1.67]	**<0.001**
	Sexuality --> Hypomania	−2.05	[−2.30, −1.80]	**<0.001**
	Sexuality --> Anxiety	−0.82	[−1.02, −0.62]	**<0.001**
	Sexuality --> Depression	−0.81	[−1.04, −0.58]	**<0.001**
Correlations	Positive coping <--> Negative coping	19.58	[17.56, 21.60]	**<0.001**
	Anxiety <--> Depression	25.12	[24.14, 26.10]	**<0.001**
	Being bullied <--> Bullying others	0.46	[0.37, 0.55]	**<0.001**
Indirect Effect	Sex --> M1 --> DV1	0.006	[−0.00, 0.01]	0.177
	Sex --> M2 --> DV1	0.10	[0.08, 0.12]	**<0.001**
	Sex --> M3 --> DV1	−0.01	[−0.014, −0.006]	**0.001**
	Sex --> M4 --> DV1	0.02	[0.01, 0.03]	**<0.001**
	Sex --> M5 --> DV1	0.07	[0.05, 0.09]	**<0.001**
	Sex --> M1 --> DV2	0.002	[−0.002, 0.006]	0.183
	Sex --> M2 --> DV2	0.05	[0.04, 0.06]	**<0.001**
	Sex --> M3 --> DV2	−0.01	[−0.014, −0.006]	**<0.001**
	Sex --> M4 --> DV2	0.005	[−0.003, 0.013]	0.231
	Sex --> M5 --> DV2	0.04	[0.03, 0.05]	**<0.001**
	Sexuality --> M1 --> DV1	0.02	[0.01, 0.03]	**<0.001**
	Sexuality --> M2 --> DV1	−0.08	[−0.09, −0.07]	**<0.001**
	Sexuality --> M3 --> DV1	−0.02	[−0.03, −0.01]	**<0.001**
	Sexuality --> M4 --> DV1	−0.02	[−0.02, −0.01]	**<0.001**
	Sexuality --> M5 --> DV1	−0.05	[−0.06, −0.03]	**<0.001**
	Sexuality --> M1 --> DV2	0.007	[0.003, 0.011]	**<0.001**
	Sexuality --> M2 --> DV2	−0.04	[−0.05, −0.03]	**<0.001**
	Sexuality --> M3 --> DV2	−0.02	[−0.03, −0.01]	**<0.001**
	Sexuality --> M4 --> DV2	−0.003	[−0.01, 0.00]	0.235
	Sexuality --> M5 --> DV2	−0.03	[−0.04, −0.02]	**<0.001**

*Note.* Sex: 0 = boys; 1 = girls. Sexuality: 0 = opposite-sex attraction; 1 = all other sexual minority groups. M1 = positive coping; M2 = negative coping; M3 = hypomania; M4 = anxiety; M5 = depression. DV1 = being bullied; DV2 = bullying other.

Bolded value <0.05, significant level was set at <0.05.

In addition, heterosexual girls reported a similar level of using positive coping, a higher level of using negative coping, a lower score on hypomania, a higher score on anxiety and depression in comparison to heterosexual boys. On the other hand, the sexual minority group reported lower usage of positive coping, higher usage of negative coping, a lower score on hypomania, anxiety and depression in comparison to opposite-sex attraction group.

## Discussion

This is the first study examining the psychological mechanism behind the pattern of being bullied and bullying others in relation to one's birth-assigned sex and sexual attraction. This study provides insights into how being bullied and bullying others are associated, increases knowledge about related factors of the victim-bully cycle, identifies the importance of coping strategies and mood states, and explores the dynamic nature of being bullying and bullying others in school settings.

### Specific socio-demographic features

The current study was composed of 2.2% same-sex-attraction girls, 1.7% same-sex-attraction boys, 7.6% both-sex-attraction-girls and 5.3% both-sex-attraction-boys out of the total sample. According to recent Chinese adolescent research, 4.1% of adolescents self-report as sexual minorities and 17.3% are unsure (Huang *et al*., 2018). Huang *et al*.'s study covered four regional areas of China, including less economically developed areas. This study was conducted in Suzhou, one of the well-developed economical cities of China. Due to the higher tolerance and friendly environment, adolescents in this study may have been more likely to disclose their sexual minority status rather than report as unsure.

The current research also shed light on a new phenomenon that has not been discussed in previous studies: there could be a number of adolescents who may not have experienced a romantic relationship or have reached a mature sexuality. In previous research, adolescents who reported being sexually attracted to neither male nor female were usually treated as asexual in their sexuality; and the number of adolescents who reported being sexually attracted to neither male nor female is usually quite small (Fenaughty *et al*., 2019). However, in this current study, 2625 boys and 2395 girls reported being sexually attracted to neither male nor female. Accordingly, new categories of sexuality were added (sexually-undeveloped boys and girls). The results showed that this subgroup of adolescents seem to be ‘protected’ from the heightened risk of being bullied or bullying others; and they also seem to report better mental health, such as lower hypomania, lower anxiety and lower depression. This may be a culturally specific phenomenon in China since Chinese parents and schools openly discourage adolescent children to be involved in romantic relationships (Li *et al*., 2010). Those adolescents who obey these suggestions tend to have a closer relationship with their parents (Li *et al*., 2010) and receive more positive feedback from school teachers. Consequently, those factors may lead to better mental health and fewer bully-related problems.

### Mood problems and bullying

It is well-documented that a sexual minority orientation is a risk factor for bullying and stressful life events (King *et al*., 2003; Berlan *et al*., 2010). Previous studies have shown that compared with heterosexual counterparts, sexual minority participants experience a significantly higher rate of bullying victimisation, with 31–33.9% being bullied *v*. 18–19.3%, respectively (Jacob *et al*., 2019; Qi *et al*., 2020). In the current results, the prevalence of bullying among sexual minorities was much lower than previous studies. It is possible that participants may feel embarrassed about bullying experiences and the lower prevalence could partly be due to underreporting. Importantly, our results confirmed our hypothesis that being bullied and bullying others are significantly correlated. In addition, a previous study indicated bully-victims may experience the greatest internal distress (Swearer *et al*., 2001). It is therefore critical that research and interventions continue to primarily focus on individuals who are being bullied and participate in the bullying others.

There is a lack of research on the association between bullying and hypomania in sexual minority individuals. A previous study in a Dutch school setting showed that both bullying and being bullied were associated with an increased risk of subclinical psychotic experiences (Horrevorts *et al*., 2014). Sexual minorities have an elevated risk of psychosis, which could be due to the experiences of social adversity such as bullying (Qi *et al*., 2020) and this association between psychological health and bullying is well-documented (Kaltiala-Heino *et al*., 2000; Bond *et al*., 2001; Fekkes *et al*., 2006; Arseneault *et al*., 2010). Our results showed that individuals with mood problems had a higher risk of being involved in bullying. Individuals with mood problems could face discriminations towards their mental health problems, which makes them more likely to be bullied. Meanwhile, individuals with mood problems have less control over their mood status, which could make them more likely to be involved in aggressive behaviours such as bullying others.

### Coping strategies and bullying

This study showed that positive coping had a significant protective effect on being bullied and a preventive effect on bullying others; negative coping was positively associated with being bullied and bullying others. In order to reduce the victim-bully cycle, it is important to coach adolescents to adopt positive coping strategies. Importantly, researchers have noted that sexual minority and heterosexual samples use different coping strategies. For example, gay men preferred to use emotional-oriented coping strategies while heterosexual men preferred to use problem-oriented coping strategies (Sandfort *et al*., 2009), with a lack of current research to understand different types of coping skills based on sexual orientation. Emotional-oriented coping strategies are associated with poorer mental health outcomes, and problem-oriented coping strategies were associated with better mental health outcomes (Sandfort *et al*., 2009). It would be useful for future research to explore the association between sexual orientation (i.e. sexual minority *v.* heterosexual) and their preferred coping strategies and bullying related information in Chinese adolescents in order to create tailored interventions for each subgroup. Furthermore, it is meaningful to design distinct forms of coping strategies between LGB groups, since sexual minority subgroups might have different needs.

### Practical implications

School-age bullying experienced by sexual orientation minorities tends to result in long-term consequences for their overall health and well-being. Recent research in England found that school-age bullying of sexual minority people is positively associated with victims' lower educational level and workplace bullying, while negatively associated with job satisfaction (Drydakis, 2019). Policies merely aimed at reducing bullying may not be effective, and additional policies are needed to promote safe and supportive school environments (Robinson and Espelage, 2012). According to a previous meta-analysis of longitudinal research, school victimisation was positively associated with students' psychological distress (Reijntjes *et al*., 2010). Many studies have emphasised the benefits of warmth, support and care for sexual minority youth (Shilo and Savaya, 2012; Hsieh, 2014; Watson *et al*., 2019). This study highlighted the importance of providing a supportive school environment to reduce bullying.

Although social support could promote positive psychosocial adjustment for sexual minority youth, the sources of social support for sexual minority youth are sparse (Button *et al*., 2012; Watson *et al*., 2019). It is important to provide social support to sexual minority youth from parents, clinicians, teachers and classmates (Watson *et al*., 2019). It is essential to provide supportive learning environments for sexual minorities, such as training teachers and staff in sexuality diversity and discussing homophobia in education (Robinson and Espelage, 2012; Robinson *et al*., 2013; Wang *et al*., 2019; Wang *et al*., 2020). In addition, in terms of school counselling, it is inappropriate to narrowly defined victims and bullies as two separate groups (Ma, 2001); the results from this study instead indicate that victims and bullies can belong to the same categories.

### Limitations

There are several limitations in the current study. First, we were unable to confirm the causality direction of the measured aspects. Second, the data are from an economically developed region of China, which may not be generalisable to all school settings in Chinese regions. Third, many other variables could affect the victim-bully cycle. Besides sexual minority personal variables, it is also critical to consider variables such as effect on school climate, discipline climate, parental and teacher involvement, academic press and school size (Ma, 2001). Future studies should aim to measure those relevant variables and establish a more comprehensive framework of the victim-bully cycle for LGB adolescents.

## Conclusion

In conclusion, this research is pioneering in exploring the interaction of being bullied and bullying others among sexual minority adolescents. We investigated the relationship between sexual orientation, mood problems, coping strategies and the victim-bully cycle. It has expanded the understanding of the possible mechanism of bullying others and being bullied among sexual minority adolescents in school settings. Unlike previous studies which investigated sexual minorities as a whole group, we identified the subgroup differences among LGB adolescents, which provided in-depth details for sexual minority adolescents. The current research highlights the importance of providing a supportive and safe learning environment for sexual minority students.

## Data Availability

The datasets used and/or analysed during the current study are available from the corresponding author on reasonable request.
